# Identification of Key Genes Involved in Seed Germination of *Astragalus mongholicus*

**DOI:** 10.3390/ijms252212342

**Published:** 2024-11-17

**Authors:** Junlin Li, Shuhong Guo, Xian Zhang, Yuhao He, Yaoqin Wang, Hongling Tian, Qiong Zhang

**Affiliations:** 1 Industrial Crop Institute, Shanxi Agricultural University, Fenyang 032200, China; 15123581751@126.com (J.L.); guo602079@163.com (S.G.); wyq784258@163.com (Y.W.); 2 School of Pharmacy, Shanxi Medical University, Taiyuan 030001, China; zdnacch@163.com; 3 College of Agriculture, Shanxi Agricultural University, Jinzhong 030801, China; 14785304219@163.com

**Keywords:** *Astragalus mongholicus*, seed germination, transcriptome, ML, WGCNA

## Abstract

Seed germination is a fundamental process in plant reproduction, and it involves a series of complex physiological mechanisms. The germination rate of *Astragalus mongholicus* (AM) seeds is significantly lower under natural conditions. To investigate the key genes associated with AM seed germination, seeds from AM plants were collected at 0, 12, 24, and 48 h for a transcriptomic analysis, weighted gene co-expression network analysis (WGCNA), and machine learning (ML) analysis. The primary pathways involved in AM seed germination include plant-pathogen interactions and plant hormone signaling. Four key genes were identified through the WGCNA and ML: Cluster-28,554.0, FAS4, T10O24.10, and EPSIN2. These findings were validated using real-time quantitative reverse transcription PCR (qRT-PCR), and results from RNA sequencing demonstrated a high degree of concordance. This study reveals, for the first time, the key genes related to AM seed germination, providing potential gene targets for further research. The discovery of N4-acetylcysteine (ac4C) modification during seed germination not only enhances our understanding of plant ac4C but also offers valuable insights for future functional research and application exploration.

## 1. Background

Seed germination is a critical stage in the plant-growth process [1,2]. Its primary purpose is to produce offspring and maintain species competitiveness in the natural environment. Additionally, it serves as a vector for the transmission of microorganisms and pathogens [3]. The appropriate temporal and spatial distribution of seed germination is essential for the survival and reproduction of seed plants [4]. The germination process begins with the uptake of water by mature, dry seeds, which leads to seed expansion and culminates in the formation of the radicle. This process encompasses a series of physiological and morphological events, including energy conversion, nutrient consumption, and changes in metabolic products [5], as well as alterations at the genetic level.

*Astragalus membranaceus* var. *mongholicus* (Bunge) P.K. Hsiao is a significant medicinal herb belonging to the Leguminosae family (*Astragalus* L.). This perennial plant is primarily found in regions such as Shanxi, Gansu, Nei Mongol, and other areas of China. Due to its remarkable pharmacological properties, the roots of *Astragalus mongholicus* (AM) are widely utilized in traditional medicine. However, wild populations of AM are becoming increasingly scarce due to overharvesting, resulting in its classification as a vulnerable species in *The Red List of Endangered Plants in China*. AM mainly propagates through seeds, but its hard seed coat poses challenges for natural breeding in the wild. Even in artificial cultivation, methods such as scarification are required to overcome the impermeability of the seed coat and increase germination rates [6]. This indicates that the seeds of Astragalus exhibit dormancy characteristics, and a low germination rate is a key internal factor affecting its cultivation success [7]. Previous studies on improving the germination rate of AM seeds have provided some insights. Conditions of both full light and complete darkness have no significant effect on the germination of AM seeds [8]. Treatment with gibberellins and hydrogen peroxide can increase the germination rate of hard Astragalus seeds [9]. Low concentrations of polyethylene glycol and NaCl also do not promote the germination of AM seeds [10]. Nevertheless, methods such as sanding, soaking in warm water, using 75% alcohol [11], and immersing in 98% concentrated sulfuric acid [12] can effectively break seed coat dormancy and improve the germination rate of AM seeds. Machine learning (ML) is a crucial branch of artificial intelligence that focuses on enabling computers to learn from data and make predictions or decisions using algorithms and statistical models. These methods can be categorized into supervised and unsupervised techniques, both of which hold significant potential for analyzing relationships in high-dimensional data [13]. Furthermore, ML is particularly effective in evaluating high-dimensional transcriptomic data and identifying biologically significant features [14,15]. In clinical settings, a weighted gene co-expression network analysis (WGCNA) is frequently combined with ML to identify diagnostic biomarkers for diseases [16,17]. However, this approach is seldom utilized to screen for genes and pathways associated with plant physiological activities.

In this study, we used a transcriptomic analysis, a WGCNA, and ML to identify key genes involved in the germination of AM seeds. By examining the regulation of these key genes and pathways throughout the germination process, we aimed to elucidate the genes and pathways implicated in AM seed germination. This research provides valuable insights into the physiological and metabolic changes that occur during the germination of *AM* seeds.

## 2. Results

### 2.1. Functional Annotation of the Transcriptome During AM Seed Germination

To investigate the mechanism of seed germination in AM, we performed RNA-Seq analysis to assess transcript-level changes across four germination stages, with each stage comprising three replicates. The raw data were derived from 12 cDNA libraries, resulting in a total of 78.87 GB of clean data, with each sample’s clean data exceeding 6 GB. The Q30 value for all samples was greater than 94% (Table S1), indicating that the high-quality transcriptome sequencing data were appropriate for further analysis. A total of 175,831 transcripts were assembled, yielding an N50 length of 2105 bp. The completeness of the transcripts was evaluated using BUSCO software (v4.0.6, Bioinformatics Group, VIB, Ghent University, Ghent, Belgium) (Figure S1). Hierarchical clustering was conducted based on the read count and expression patterns of the transcripts aligned with Corset. The longest cluster sequence obtained from the hierarchical clustering was designated as an unigene for subsequent analysis, resulting in a total of 103,152 unigenes with an N50 of 2341 bp (Tables S2 and S3). To acquire comprehensive information on the assembled transcriptome, similarity searches were performed against the Kyoto Encyclopedia of Genes and Genomes (KEGG), Non-redundant Protein Sequence Database (Nr), SwissProt, Gene Ontology (GO), Eukaryotic Orthologous Groups of Proteins (KOG), Translated EMBL Nucleotide Sequence Data Library (TrEMBL), and Protein Families Database (Pfam) databases, using a significance threshold of E ≤ 10^−5^ and the BLAST algorithm. Among these, 60,024 sequences (58.19% of the total) were annotated in KEGG, 77,698 (75.32%) in Nr, 56,918 (55.18%) in SwissProt, 67,948 (65.87%) in GO, 48,784 (47.29%) in KOG, 77,633 (75.26%) in TrEMBL, and 52,232 (50.64%) in Pfam (Table S4).

### 2.2. Differential Gene Expression and Enrichment Analysis

**Figure 1:**
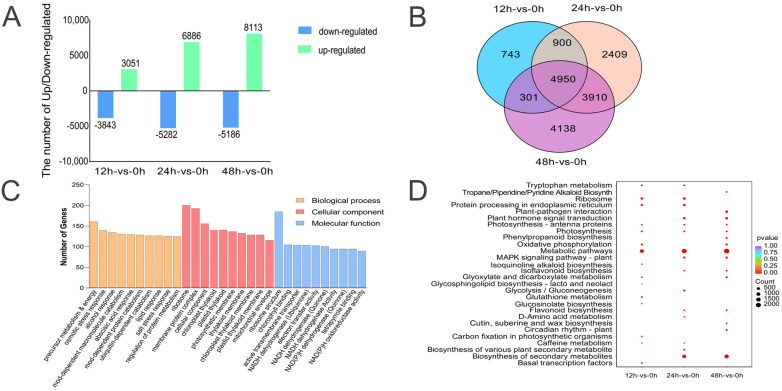
DEGs during germination of AM seeds. (**A**) Upregulation and downregulation of DEGs at 12 h, 24 h, and 48 h, respectively, compared to 0 h. (**B**) Venn diagram illustrating DEGs. (**C**) GO enrichment analysis of DEGs. (**D**) KEGG enrichment analysis.

The DESeq2 package (v1.22.2, Bioconductor, Seattle, WA, USA) was employed for differential gene expression analysis. Using the criteria of |log2 Fold Change| ≥ 1, FDR < 0.05, and *p*-value < 0.01, we compared all upregulated and downregulated genes. As the duration of seed germination increased, the number of differentially expressed genes (DEGs) gradually rose (Figure 1A). Venn diagrams comparing the time points of 12 h-vs-0 h, 24 h-vs-0 h, and 48 h-vs-0 h indicated that 4950 genes exhibited significant changes throughout the germination process (Figure 1B). GO enrichment analysis of DEGs across the four germination stages revealed that these genes were broadly distributed among three functional categories: biological process, molecular function, and cellular component (Figure 1C). In the biological process category, the majority of genes were involved in the generation of precursor metabolism and energy, indicating that the transition from dormancy to an active growth state necessitates extensive breakdown of seed-storage materials for energy and precursor metabolites. This process supports cell growth and division, ultimately driving seedling emergence and growth. In the cellular component category, most genes were localized to the ribosome, underscoring the critical role of protein synthesis during seed germination, as newly synthesized proteins were essential for reestablishing cellular functions and promoting seedling growth. In the molecular function category, many genes were associated with the mitochondrial envelope, highlighting the importance of mitochondrial activation for energy production during seed germination. This underscored the active involvement of mitochondria in regulating intracellular energy balance and metabolic activities, which ensured a continuous energy supply for seed germination. Together, these findings emphasized the crucial role of mitochondria in supporting the energy needs necessary for successful germination.

KEGG enrichment analysis of the DEGs revealed significant enrichment in metabolic pathways across the three time points, indicating that these pathways were highly active during seed germination. This finding demonstrated that metabolic regulation, which encompasses the breakdown of storage materials, energy generation, and the synthesis of new cellular components, plays a crucial role in the germination of AM seeds (Figure 1D).

### 2.3. Identification of Coexpressed Gene Modules Associated with Seed Germination in AM

**Figure 2:**
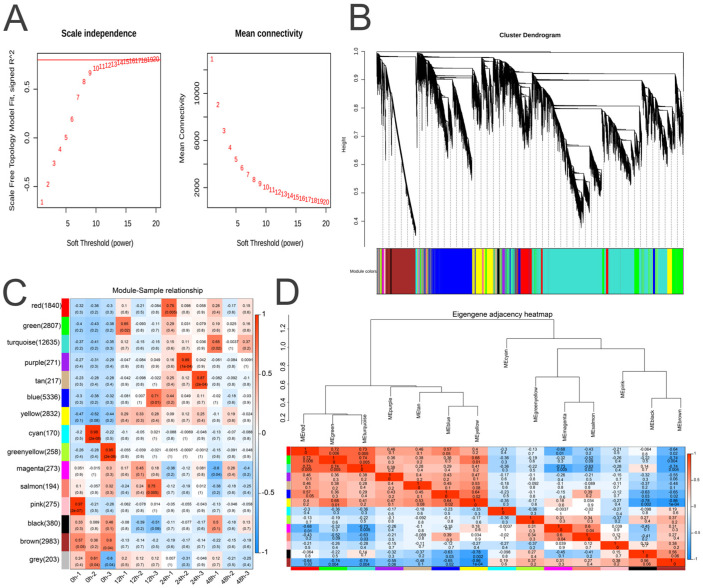
Expression network analysis of genes related to AM seed germination. (**A**) Appropriate soft thresholds were established to construct the scale-free network. (**B**) The cluster dendrogram illustrates the results of hierarchical clustering among genes, with different modules indicated by distinct colors at the bottom of the figure. Each module represents a set of highly co-expressed genes. (**C**) The module–sample relationship illustrates the correlation between various gene modules and sample features. (**D**) The eigengene adjacency heatmap displays the similarity between genes characterized by their respective modules.

To understand the biological processes involved in the germination of AM seeds from a holistic network perspective, we conducted a WGCNA (Figure 2). The pick soft threshold function in the WGCNA package (version 1.71, Peter Langfelder and Steve Horvath, University of California, Los Angeles, CA, USA) in R was utilized to calculate the optimal power value, using an R-squared cut-off of 0.85, which resulted in an appropriate soft threshold of 20 (Figure 2A). The WGCNA categorized the transcripts obtained from sequencing into 15 distinct modules. The majority of transcripts were distributed in the blue and turquoise modules, with fewer transcripts found in the tan and salmon modules (Figure 2B,C). In various stages of seed development, gene expression in different modules shows significant correlations with specific stages as follows: the dry seed stage (0 h-1, 0 h-2, and 0 h-3) shows significant upregulation of the pink, cyan, and green yellow modules, indicating a high expression of genes within these modules during this phase. In the seed imbibition stage (12 h-1, 12 h-2, and 12 h-3), the green, blue, and salmon modules are significantly upregulated, suggesting active gene expression in these modules during imbibition. During the seed-germination stage (24 h-1, 24 h-2, and 24 h-3), the red, purple, and tan modules exhibit significant upregulation. In the seed swelling stage (48 h-1, 48 h-2, and 48 h-3), the turquoise module is significantly upregulated, indicating substantial gene expression in this module during the swelling phase (Figure 2C,D). We performed a correlation analysis between MM and GS for the 15 modules. We selected modules with cor > 0.7 and *p*-value < 0.05 for further analysis. This resulted in the selection of the grey module (cor = 0.93, *p* = 3 × 10^−103^), purple module (cor = 0.93, *p* = 3 × 10^−52^), tan module (cor = 0.94, *p* = 4.1 × 10^−39^), magenta module (cor = 0.74, *p* = 5 × 10^−38^), and green module (cor = 0.74, *p* = 6.4 × 10^−199^), totaling 3771 genes (Figure S2).

### 2.4. Functional Enrichment Analysis of Key Module Genes

**Figure 3:**
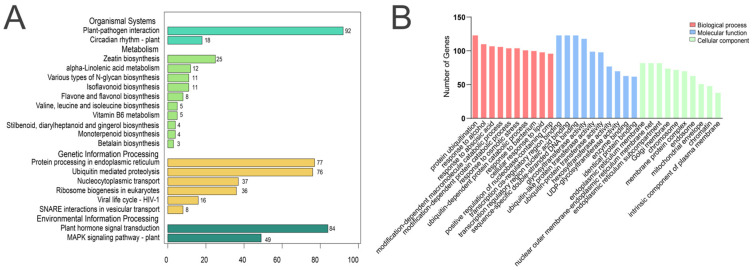
KEGG and GO analysis of module genes related to AM seed germination. (**A**) Key module KEGG pathway analysis: The horizontal axis represents the number of genes enriched in the top 20 pathways, while the vertical axis indicates the names of the KEGG pathways. (**B**) Key module GO function analysis: The horizontal axis displays the names of the GO entries, and the vertical axis represents the number of genes enriched in the top 10 GO functions.

The WGCNA method aims to identify co-expressed gene modules and explore the associations between gene networks and target traits of interest. By conducting enrichment analysis on the genes within key modules, detailed insights can be obtained [18]. During seed germination, a total of 138 pathways were identified across five key modules. Among these, the plant-pathogen interaction (*n* = 92) and plant hormone signal transduction (*n* = 84) pathways exhibited the highest levels of gene enrichment. Additionally, protein processing in the endoplasmic reticulum (*n* = 77) and ubiquitin-mediated proteolysis (*n* = 76) were also significantly enriched (Figure 3A). GO enrichment analysis revealed that, in the biological process category, key module genes were primarily involved in protein ubiquitination (*n* = 123), response to alcohol (*n* = 110), and response to abscisic acid (*n* = 107). In the cellular component category, these genes were predominantly located in the endoplasmic reticulum membrane (*n* = 82) and the nuclear outer membrane-endoplasmic reticulum membrane net (*n* = 82). In the molecular function category, the primary activities involved transcription cis-regulatory region binding (*n* = 123) and transcription regulatory region nucleic acid binding (*n* = 123). (Figure 3B). The key module genes were predominantly enriched in the plant-pathogen interaction and plant hormone signal transduction pathways, indicating that the germination stage of AM seeds was particularly sensitive to external environmental factors. In contrast, processes such as ubiquitin-mediated proteolysis and ribosome biogenesis underscored the complexity and critical nature of the internal regulatory mechanisms governing seed germination.

### 2.5. Screening of Feature Genes

**Figure 4:**
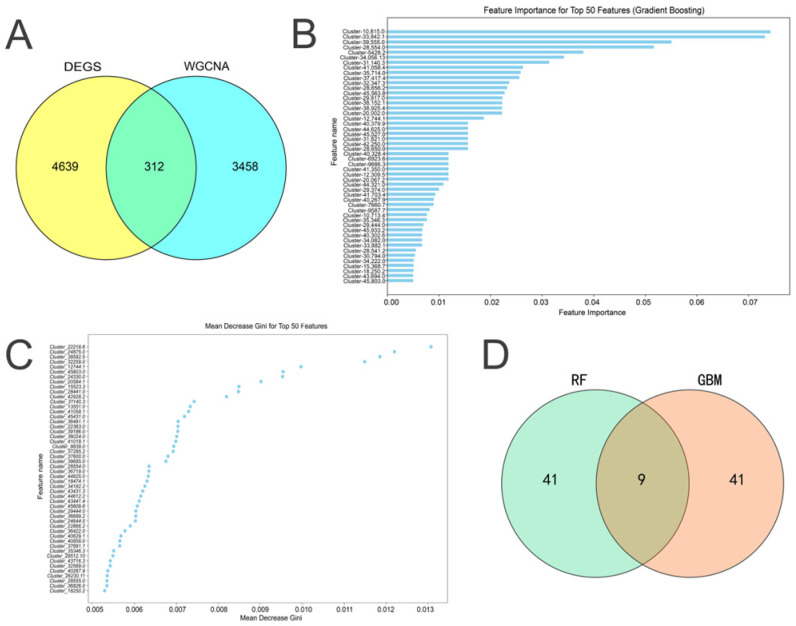
Feature gene selection for AM seed germination. (**A**) DEGs and WGCNA of the overlapping screened genes. (**B**) GBM screening of the characterized genes, with the horizontal axis representing the character importance score and the vertical axis representing the gene name. (**C**) RF algorithm screening of the characterized genes, with the horizontal axis indicating the average Gini index decline value and the vertical axis indicating the gene name. (**D**) Feature genes identified from RF and GBM screening.

A Venn diagram analysis (Figure 4A) was performed to compare the five module genes (*n* = 3771) with the differentially expressed genes (DEGs, *n* = 4950), identifying 312 overlapping genes, of which 293 were upregulated and 19 were downregulated. To further select feature genes related to AM seed germination, gradient boosting machine (GBM, Figure 4B) and random forest (RF, Figure 4C) algorithms were employed. In GBM, importance scores for each gene were calculated based on its contribution to all decision trees. By analyzing these scores, we identified the most influential genes that significantly affect prediction outcomes [19]. In addition, RF is a widely used machine learning method for feature selection, which assesses the importance of each gene by calculating the reduction in the Gini index (for classification) or the mean squared error reduction (for regression) at tree nodes. This method is effective at identifying features that contribute most to the prediction results, and it is particularly suitable for high-dimensional data. RF also handles multicollinearity well, which is crucial for analyzing gene expression data. Furthermore, by introducing randomness, RF reduces overfitting and enhances model generalizability [20,21]. Using these two machine learning methods, we selected nine feature genes (Table S5, Figure 4D) from the 312 overlapping genes. These feature genes, which performed well in both the GBM and RF models, represent genes with high predictive power and biological relevance, providing important candidates for further research.

### 2.6. Analysis of Key Genes

**Figure 5:**
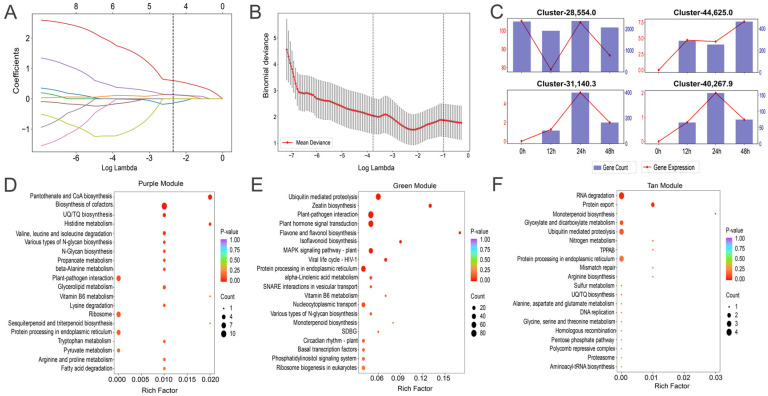
Key genes involved in AM seed germination. (**A**) LASSO coefficient path diagram. The horizontal axis represents the log λ value, while the vertical axis displays the regression coefficient of the gene. At the optimal λ value (indicated by the vertical dashed line in the figure), the LASSO method identifies the key genes. (**B**) Cross-validation error plot of LASSO. The horizontal axis shows the log λ value, and the vertical axis represents the mean deviation. The red solid line indicates the mean deviation, while the gray shading represents the standard error. The vertical dashed line marks the optimal λ value, which corresponds to the smallest error. (**C**) Plot of expression changes of the four key genes at different time points. The horizontal axis denotes the time points, the vertical axis indicates gene expression on the left side, and gene counts on the right side. The bar graph represents gene counts, and the line graph illustrates gene expression. (**D**) KEGG pathway enrichment analysis graph for the purple module. (**E**) KEGG pathway enrichment analysis graph for the green module. (**F**) KEGG pathway enrichment analysis for the tan module.

After identifying the nine feature genes from the intersection of GBM and RF (Figure 4D), least absolute shrinkage and selection operator (LASSO) was employed to pinpoint four key genes (Table S6; Figure 5A,B): Cluster-28,554.0, Cluster-31,140.3, Cluster-44,625.0, and Cluster-40,267.9. Among these genes, Cluster-31,140.3, Cluster-44,625.0, and Cluster-40,267.9 were upregulated during AM seed germination, while Cluster-28,554.0 was downregulated. In this experiment, 0 h was used as the control group, and 12, 24, and 48 h were used as the experimental groups. The changes of these four key genes were as follows: the expression level of Cluster-44,625.0 showed a significant upward trend, while the expression levels of Cluster-31,140.3 and Cluster-40,267.9 began to decrease significantly after 24 h, decreasing by 38.5% and 45.0%, respectively. Cluster-28,554.0 first decreased, then increased during seed germination, and then decreased again (Figure 5C).

The identification of overlapping genes and the application of multiple ML algorithms in this study resulted in the selection of four key genes from the feature set. According to the WGCNA module classification, Cluster-31,140.3 was part of the purple module, which was primarily associated with the biosynthesis of cofactors, pantothenate, and CoA biosynthesis (Figure 5D). Both Cluster-28,554.0 and Cluster-44,625.0 belonged to the green module, which was mainly related to plant-pathogen interaction, ubiquitin-mediated proteolysis, and zeatin biosynthesis (Figure 5E). Cluster-40,267.9 was categorized within the tan module, which was primarily involved in RNA degradation, protein export, and ubiquitin-mediated proteolysis (Figure 5F). Through the use of LASSO and the WGCNA, this study identified four key genes (Cluster-28,554.0, Cluster-31,140.3, Cluster-44,625.0, and Cluster-40,267.9) that play a role in AM seed germination. A detailed annotation of the expression changes and functions of these genes highlighted their important roles in various biological processes, providing valuable insights and a foundation for further research into the mechanisms underlying AM seed germination.

To further elucidate the functions of the four key genes, we analyzed their annotation information. Cluster-28,554.0 encodes a protein of unknown function, and our understanding of its role remains limited. However, since both Cluster-28,554.0 and Cluster-44,625.0 are components of the green module, it is hypothesized that these two genes may exhibit similar functions. Cluster-31,140.3 encodes the ATP-dependent RNA helicase DEAH13, also known as FAS4 (*At1g33390*). This gene encodes a protein that contains an HA2 domain associated with RNA helicases and is a member of the DEAH-box RNA helicase family. Cluster-40,267.9 encodes RNA 4-methylcytidine acetyltransferase 1, with the coding gene identified as T10O24.10 (*At1g10490*). This protein is generally associated with the Cyclin N-terminal domain, which is linked to cell cycle regulation. It plays a crucial role in ribosome biogenesis by specifically catalyzing the formation of N4-acetylcysteine (ac4C) in 18S rRNA and tRNA. The synthesis of N4-acetylcysteine is essential for plant growth and development [22]. Cluster-44,625.0 encodes the protein EPSIN2/EPN2 (*At2g43160/At2g43170*), also known as the balanced nucleotide transporter, which contains an ENTH (epsin N-terminal homology) domain, plays a significant role in protein trafficking, particularly in endocytosis, and is involved in ribosome biogenesis in eukaryotes according to KEGG metabolic pathway annotations. Seed germination is a complex and dynamic process, and the four key genes identified interact to ensure successful seed germination and early growth.

### 2.7. qRT-PCR Validation of Key Genes

**Figure 6:**
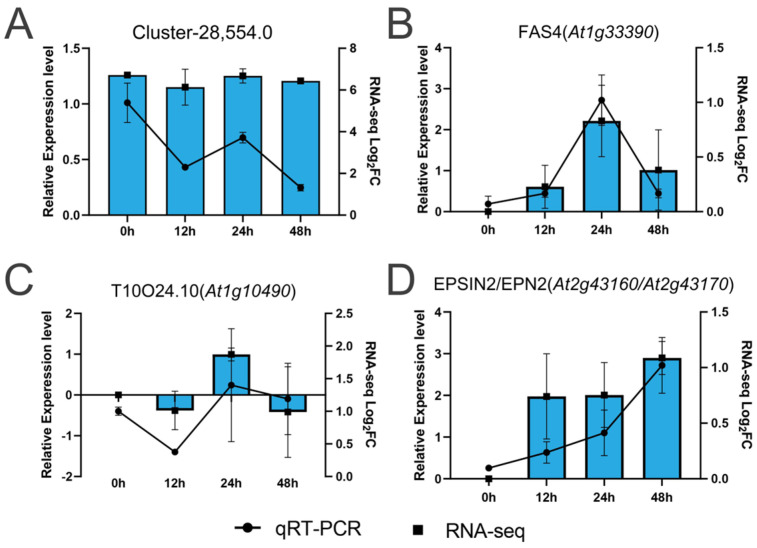
qRT-PCR validation of four key genes. The black lines represent the qRT-PCR results for the key genes, while the blue bars indicate the RNA-seq values. Each sample was analyzed with three biological replicates for qRT-PCR. Error bars represent the standard deviation of the relative expression levels from the three biological replicates. (**A**) Expression level of Cluster-28,554.0 in RNA-Seq and qRT-PCR validation results; (**B**) Expression level of FAS4 in RNA-Seq and qRT-PCR validation results; (**C**) Expression level of T10O24.10 in RNA-Seq and qRT-PCR validation results; (**D**) Expression level of EPSIN2/EPN2 in RNA-Seq and qRT-PCR validation results.

The expression levels of key genes were validated using qRT-PCR (Figure 6). The results indicated that the expression patterns of the four selected key genes were generally consistent with the RNA-Seq data, confirming a strong correlation between the RNA-Seq and qRT-PCR results.

## 3. Discussion

Seed germination is a complex process governed by the interaction of numerous intrinsic factors [23]. Understanding the entire germination process, from initial water uptake to radicle emergence, is essential for gaining comprehensive insights into this developmental stage. Accurately determining the transition time from water uptake to radicle emergence during seed germination is crucial [24]. Previous studies have shown that seed imbibition occurs in three distinct phases: Phase I, which lasts for 6 h from the onset of water absorption; Phase II, which consists of a plateau lasting until 24 h; and Phase III, which culminates in the initiation of germination [25]. AM seeds exhibit rapid water absorption within the first 1 to 12 h, followed by a gradual approach to saturation from 12 to 24 h [26]. For AM, the germination phase lasts from 0 to 2 days, while the post-germination growth phase extends from 3 to 8 days [27]. In this study, transcriptomic analyses of AM seeds at 0 h, 12 h, 24 h, and 48 h after germination revealed significant KEGG/GO enrichment, indicating that seed germination requires substantial energy for growth and development. GO enrichment analysis highlighted the generation of precursor metabolites and energy as a key functional category, underscoring the fact that energy metabolism is a central process in seed germination. Additionally, KEGG pathway analysis indicated enrichment in metabolic pathways, demonstrating that metabolic activities are continuously adjusted and activated during the transition from seed dormancy to active growth. Correlation analysis between MM and GS across 15 modules demonstrated five key modules with cor > 0.7 and *p*-value < 0.05. Subsequent KEGG and GO enrichment analyses revealed that these five key modules were predominantly associated with the plant–pathogen interaction and plant hormone signal transduction pathways. These findings indicate that these two pathways exhibit heightened activity throughout the seed-germination process in AM.

The plant–pathogen interaction pathway includes genes associated with plant defense mechanisms and immune responses [28]. This indicates that, during the seed germination phase, seeds are particularly susceptible to pathogen attacks, and the genes enriched in this pathway play crucial roles in pathogen recognition, the initiation of defense responses, and signaling processes that bolster seed resistance to diseases. Additionally, the majority of these genes are also enriched in the plant hormone signal transduction pathway, indicating that precise regulation of plant hormone synthesis, secretion, and signaling is vital for seed germination, influencing cellular division, expansion, and developmental growth [29]. A WGCNA provides a visual representation of the complex co-expression patterns among genes, facilitating the identification of module genes [30]. Furthermore, ML algorithms can enhance the identification of key genes within these modules, improving the accuracy and reliability of the gene selection process [29,31]. Seed germination is a complex and highly coordinated process that requires precise gene regulation and an adequate energy supply. In this study, we identified four key genes (Cluster-28,554.0, FAS4, EPN2, and T10O24.10) through a WGCNA and three ML models. Among these, the functional understanding of Cluster-28,554.0 is limited due to a lack of relevant studies. RNA helicases represent a large and complex gene family involved in various aspects of RNA metabolism [32]. RNA helicases are frequently associated with ribonucleoprotein complexes, which are essential for ribosome assembly, degradation, and the regulation of translation. Notably, FAS4, an ATP-dependent RNA helicase, regulates reproductive development through sub functionalization, which is critical for plant reproduction [33]. Studies have shown that FAS4 may be expressed at specific stages of plant development or under certain developmental and environmental conditions [34]. The expression of FAS4 during the germination of AM indicates its specific role during this critical growth phase. Ac4C is a conserved modification found in rRNA and tRNA [35,36,37]. Among various mRNA modifications, ac4C is unique because of its acetylation. In human cell lines, ac4C enhanced mRNA stability and translation initiation [38]. However, the existence, distribution patterns, and potential functions of ac4C modifications in plants remain largely unexplored. Studies have shown that ac4C was enriched at translation initiation sites in rice mRNA and at both initiation and termination sites in Arabidopsis mRNA [39]. It is hypothesized that ac4C modification may promote mRNA stability in plants, although extensive research is needed to elucidate its underlying mechanisms [40]. In the transcriptome of AM seeds, the enzyme RNA 4-methylcytidine acetyltransferase 1, which catalyzes the formation of ac4C in 18S rRNA, was identified [22]. Furthermore, its gene expression levels gradually increase during germination, indicating significant production of ac4C during seed germination and its crucial regulatory role in this process. Specifically, the increase in ac4C modification may stabilize key mRNAs, facilitate their translation, and ensure the efficient synthesis of proteins required for seed germination. This dynamic modification further emphasizes the crucial function of ac4C in regulating gene expression and facilitating seed germination and early growth. EPSIN proteins are an evolutionarily conserved family of membrane proteins that play crucial roles in endocytosis and signal transduction [41]. This protein family has been primarily studied in humans and animals [42]. Evidence from mouse models showed that knockout of EPSIN1 and EPSIN2 leads to embryonic lethality, highlighting their critical functions in embryonic development [43]. Similarly, during the process of seed germination in AM, EPSIN2 expression may play an important role in early developmental stages to ensure proper embryonic development and successful seed germination.

## 4. Materials and Methods

### 4.1. Materials

The AM seeds used in this study were obtained from the Economic Crop Research Institute of Shanxi Agricultural University (37°24′05″ E, 111°78′65″ N). The seeds were identified as AM by researcher Tian H from Shanxi Agricultural University. The HQ-233 seeds demonstrated excellent field-germination performance. All experimental seeds were harvested from the same AM plant in 2023 and stored in a seed bank at 4 °C. The seeds were disinfected with a 5% sodium hypochlorite solution for 5 min, rinsed 6–7 times with running water (with each rinse lasting 1–2 min), soaked in boiling water for 60 s, and then removed and placed in Petri dishes for cultivation. The treated seeds were incubated in a greenhouse at a temperature of 20–25 °C until germination.

**Figure 7:**
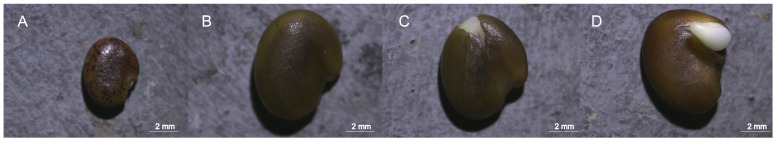
Morphological characteristics of AM seed germination at four stages: (**A**) Seed dormancy (0 h). (**B**) Seed water absorption and swelling (12 h). (**C**) Seed coat dehiscence (24 h). (**D**) Radicle breakthrough (48 h).

This study focused on four stages of seed germination: 0 h (seeds without imbibition), 12 h (seeds fully imbibed with water), 24 h (seed coat cracking stage), and 48 h (radicle emergence stage) (Figure 7). The seeds were immediately frozen in liquid nitrogen and stored at −80 °C for subsequent analysis.

### 4.2. Sequencing Results

The RNA from the AM seeds was extracted using the CTAB-PBIOZOL method. The extracted RNA was dissolved in 50 µL of DEPC-treated water. Total RNA was subsequently identified and quantified using a Qubit 4.0 (Thermo Fisher Scientific, Waltham, MA, USA) fluorometer and a Qsep400 (BiOptic, New Taipei City, Taiwan) high-throughput bio-fragment analyzer. Most eukaryotic mRNAs possess a poly(A) tail, which was utilized to enrich poly(A)-tailed mRNAs with oligo(dT) magnetic beads for mRNA library construction. The libraries were sequenced on the Illumina NovaSeq 6000 platform at Wuhan MetWare Biotechnology Co., Ltd., Wuhan, China. Following sequencing, the raw data underwent several quality control steps, including data filtering, assessment of sequencing error rates, and examination of GC content distribution. Reads containing adapters, reads with more than 10% N content, and reads with over 50% of bases having a quality score (Q) ≤ 20 were removed to obtain clean reads for subsequent analysis (Table S1). The clean reads were assembled into transcripts using Trinity (v2.13.2, Trinity Software, Washington, DC, USA) [44]. The assembled transcripts were then clustered and deduplicated using Corset (https://github.com/Oshlack/Corset) (accessed on 25 July 2024) to refine the dataset for further analysis.

After filtering the raw sequencing data, high-quality reads were obtained. These reads were assembled into transcript sequences for the species using Trinity. The transcripts were then deduplicated to generate unigene sequences with Corset. The high-quality reads were aligned to the deduplicated transcriptome to calculate gene expression levels. Transcript expression levels were determined using RSEM software(v1.3.1, RSEM Software, University of California, Berkeley, CA, USA), and FPKM for each transcript was calculated using transcript length (Figure S3). To predict the potential functions and biological pathways of the genes, DIAMOND [45] BLASTX software (v2.13.0, NCBI, Bethesda, MD, USA) was employed to align unigene sequences against the KEGG, NR, Swiss-Prot, GO, KOG, and TrEMBL databases. After predicting the amino acid sequences of the unigene, HMMER software (v2.13.0+, NCBI, Bethesda, MD, USA) was utilized for alignment with the Pfam database to obtain unigene annotation information (Tables S4 and S7).

### 4.3. Screening and Analysis of DEGs

Differential expression analysis between sample groups was conducted using DESeq2 (version 1.22.2, Bioconductor, Seattle, WA, USA) [46,47], which identified differentially expressed gene sets between two biological conditions. The Benjamini–Hochberg method was employed to adjust *p*-value for multiple hypothesis testing, with corrected *p*-value and |log2 fold change| serving as thresholds for significant differential expression. The criteria for identifying differentially expressed genes were |log2 Fold Change| ≥ 1 and false discovery rate (FDR) < 0.05. Differentially expressed genes were annotated for categories, functions, and pathways using the Gene Ontology (GO) database (http://geneontology.org/) (accessed on 25 July 2024) and the Kyoto Encyclopedia of Genes and Genomes (KEGG) database (http://www.kegg.jp/ or http://www.genome.jp/kegg/) (accessed on 25 July 2024).

### 4.4. WGCNA and ML

A WGCNA is advantageous for studying gene set expression. The WGCNA R package was employed in subsequent stages to construct and modularize various gene networks. The samples were clustered to identify any potentially significant outliers that may exist. Following this, a co-expression network was established using an automated network system. The “WGCNA” package in R software (v4.3.3, R Foundation for Statistical Computing, Vienna, Austria) was utilized for constructing and visualizing the network [48], with the following parameters: mergeCutHeight = 0.25, RsquaredCut = 0.85, TOMType = “signed” and minModuleSize = 50. MM is correlated with GS, and colors are selected for further analysis based on a correlation coefficient (cor) > 0.7 and a *p*-value < 0.05.

### 4.5. Key Genes

RF is a machine learning method that utilizes decision trees to assess variable importance by scoring the significance of each variable [49]. GBM is an ensemble learning algorithm that evaluates the contribution of each input feature to the prediction outcome, with more important features exerting a greater influence on the predictive results [19]. LASSO regression is another machine learning technique that performs variable selection and complexity regularization while fitting a generalized linear model, the scikit-learn package (v0.24.2, scikit-learn developers, USA) in python (v3.12, Python Software Foundation, Beaverton, OR, USA) is commonly used. The degree of complexity adjustment in LASSO is governed by the parameter *lambda*. A larger *lambda* value imposes a greater penalty on linear models with more variables, resulting in fewer selected variables and more representative genes [50]. This study employed these three machine learning models. Initially, feature genes were screened using the GBM and RF algorithms, followed by the selection of key genes using LASSO. 

### 4.6. Real-Time Quantitative Reverse Transcription PCR (qRT-PCR)

RNA was extracted using the Plant RNA Extraction Kit (DP432) following the manufacturer’s instructions. Transcript-specific primers were designed with Primer 3 (Table S8) and synthesized by Shanghai Yuying Biotechnology Co. (Shanghai, China). The synthesized primers were subsequently optimized (Figures S4 and S5). qRT-PCR was conducted using Power qPCR PreMix (GENEray, GK8020, Guangzhou, Guangdong, China) on the CFX384 Touch™ Real-Time PCR Detection System (Bio-Rad Laboratories, Hercules, CA, USA) under the following conditions: 95 °C for 10 min, followed by 40 cycles of 95 °C for 10 s and 60 °C for 34 s. The 18S RNA served as an internal reference, and relative expression levels were calculated using the 2^−ΔΔCt^ method [51]. Each biological sample was analyzed in triplicate.

### 4.7. Statistical Analysis

Data analysis was conducted using Microsoft Excel 2019 (Microsoft Corporation, Redmond, WA, USA) for initial data processing and organization. A WGCNA was performed using the “WGCNA” package in R software (v4.3.3, R Foundation for Statistical Computing, Vienna, Austria). Data preprocessing and analysis were executed using the Python programming language. The Pandas library facilitated data cleaning and manipulation, including handling missing values with the “dropna” and “fillna” functions, performing data formatting and calculations with the “apply” and “map” functions, and filtering data based on specific conditions using the “query” function. Additionally, the NumPy library was employed for data analysis and computation. Statistical analysis and regression model construction were carried out using the scikit-learn library. Data visualization was performed in a Python environment utilizing the matplotlib and seaborn libraries. Machine learning model development and evaluation were conducted in Python, also using the scikit-learn library. Feature selection was executed with the “SelectKBest” function, and classification models were developed using the random forest classifier. Model performance was assessed through 5-fold cross-validation.

## 5. Conclusions

Seed germination is a complex and highly coordinated process that necessitates precise gene regulation and an adequate energy supply. Transcriptomic analyses have demonstrated the reliance of seeds on energy metabolism during germination and highlighted the central roles of several key genes in various biological processes. Our study identified four key genes—Cluster_28554.0, FAS4, EPN2, and T10O24.10—as crucial regulators of the germination process. These findings not only enhance our understanding of the mechanisms underlying AM seed germination but also offer valuable insights for further functional research and for the regulation of plant growth.

## Data Availability

The data and pictures are in the Supplementary Materials (https://zenodo.org/records/13927134 at 10.5281/zenodo.13927134), as well as the transcribe raw data can be found in the NCBI database (https://www.ncbi.nlm.nih.gov/sra/?term=PRJNA1165939).

## Supplementary Materials

The following supporting information can be downloaded at: https://www.mdpi.com/article/10.3390/ijms252212342/s1.

## Abbreviations

WGCNA	Weighted Gene Coexpression Network Analysis
ML	Machine Learning
FPKM	Fragments Per Kilobase of Transcript Per Million Mapped Reads
GO	Gene Ontology
KOG	Eukaryotic Orthologous Groups of Proteins
TrEMBL	Translated EMBL Nucleotide Sequence Data Library
KEGG	Kyoto Encyclopedia of Genes and Genomes
Pfam	Protein Families Database
Nr	Non-redundant Protein Database
MM	Module Membership
GS	Gene Significance
RF	Random Fores
GBM	Gradient Boosting Machine
LASSO	Least Absolute Shrinkage and Selection Operator
SDBG	Stilbenoid, diarylheptanoid and gingerol bisynthesis
UQ/TQ biosynthesis	Ubiquinone and other terpenoid-quinone biosynthesis
TPPAB	Teopane, pineridine and pyridine alkaloid biosynthesis
