# Supplementary material for: Identification of Key Genes Involved in Seed Germination of Astragalus mongholicus
Source: Int J Mol Sci. 2024 Nov 17;25(22):12342. doi: 10.3390/ijms252212342 (PMC11595215; doi:10.3390/ijms252212342)
Supplement: Supplementary file 1 [file ijms-25-12342-s001.zip › ijms-3169640- Figure Supplementary (Figure S1 -S5).pdf]

Figure of Contents:

Figure S1 BUSCO Assessment Results

Figure S2 MM-GS plot

Figure S3 Boxplot of expression

Figure S4 The key and 18 s internal genes amplification curve

Figure S5 Melt curves of the 18S reference and key genes

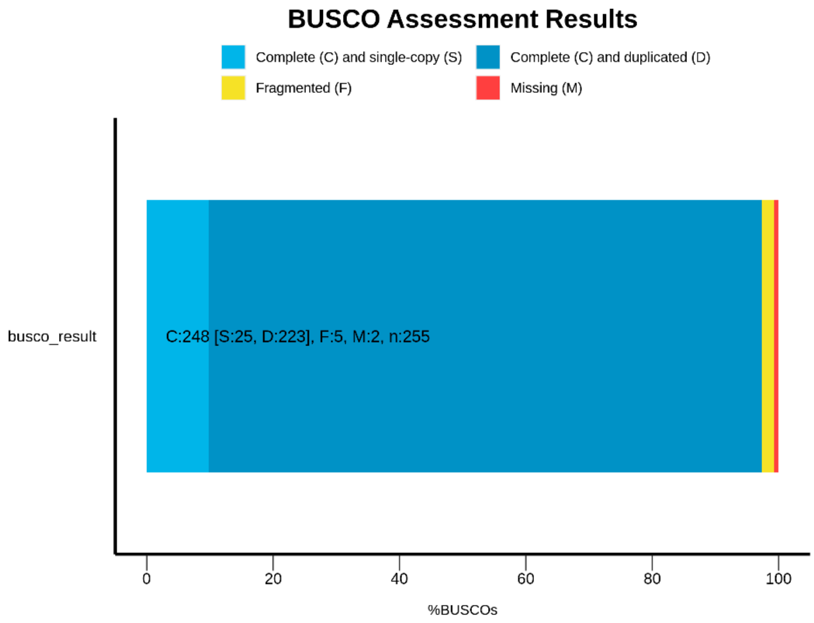

Figure S1 BUSCO Assessment Results

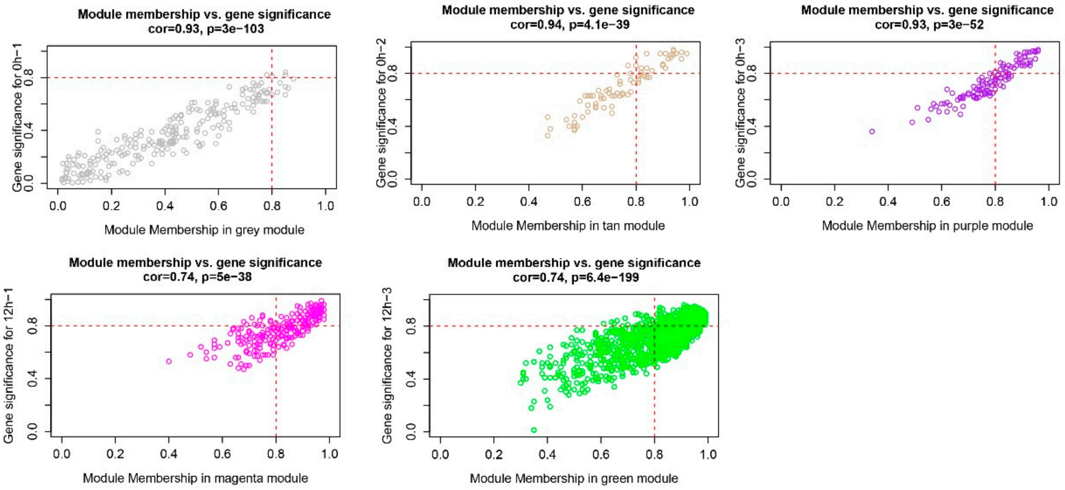

Figure S2 MM-GS plot

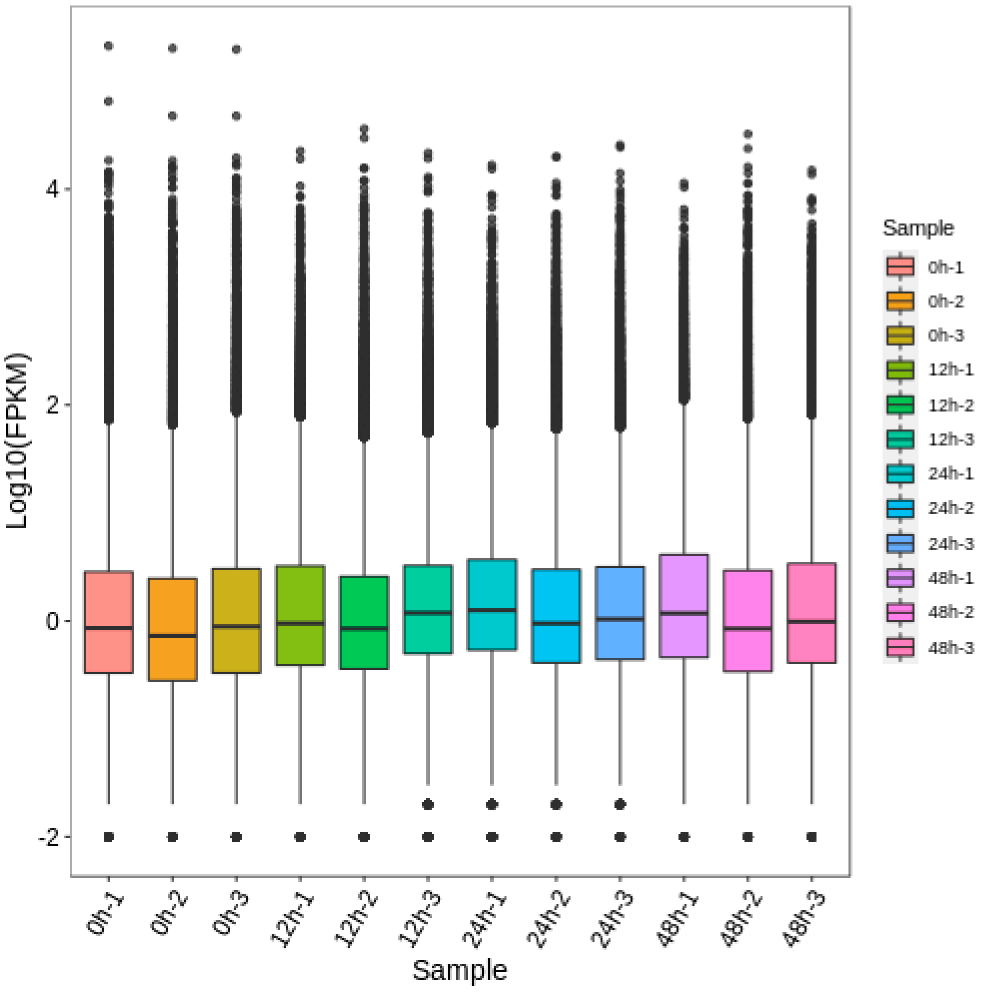

Figure S3 Boxplot of expression

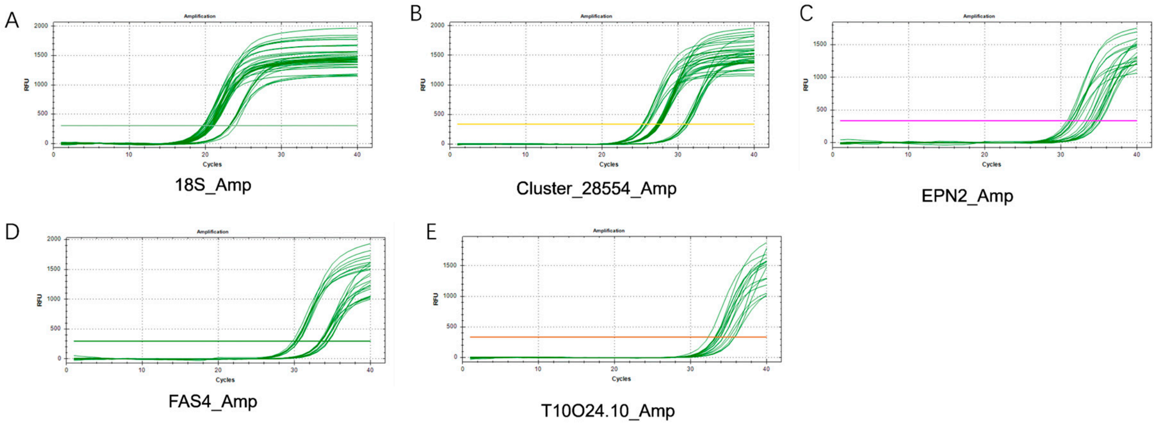

Figure S4 The key and 18 s internal genes amplification curve

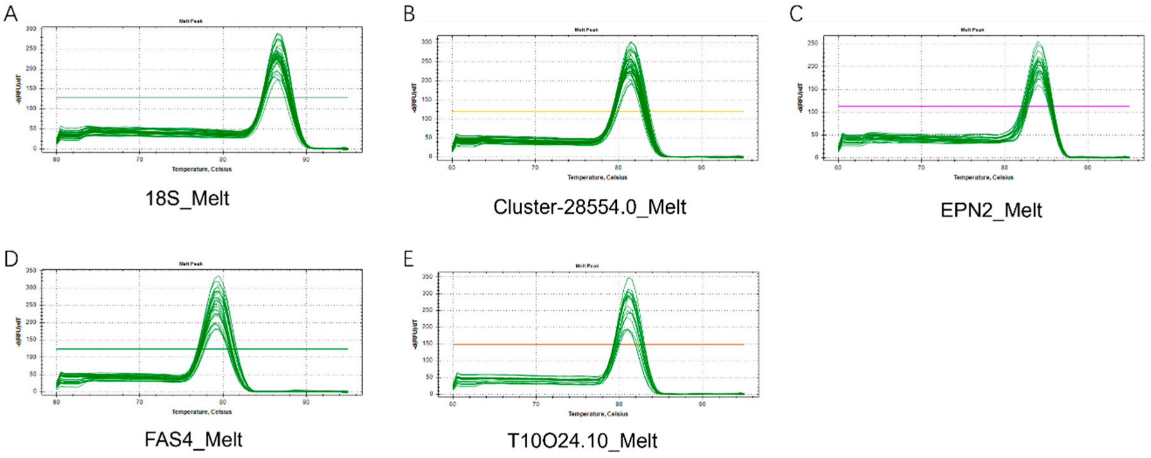

Figure S5 Melt curves of the 18S reference and key genes
